# On the necessity of complete caries excavation: a narrative review

**DOI:** 10.1186/s13005-025-00573-y

**Published:** 2025-12-12

**Authors:** Till Dammaschke

**Affiliations:** Department of Periodontology and Operative Dentistry, University of Münster, Waldeyerstrasse 30, Münster, 48149 Germany

**Keywords:** Complete caries excavation, Selective caries excavation, Pulp capping, Vital pulp therapy, Review

## Abstract

Selective caries excavation is recommended as an alternative to complete (non-selective) caries excavation. In the selective procedure, a thin layer of caries-altered dentine is left in deep cavities to avoid pulp exposure and the tooth is restored with a definitive bacteria-proof (sealed) restoration in the same appointment. The theory behind selective caries excavation is that leaving carious dentine in deep lesions is not a problem, because the cavity is sealed by an adhesive restoration. Thus, the remaining bacteria are cut off from any substrate, “starve”, and the caries cannot progress. However, not only cariogenic bacteria, which are dependent on a supply of substrate in the form of carbohydrates, occur in carious dentine, but also anaerobic asaccharolytic bacteria, which can metabolise proteins from demineralized collagen. These bacteria remain active even under adhesive restorations and lead to inflammation of the pulp tissue. In addition, endotoxins, which are released by decomposed (“starved”) microorganisms, diffuse through the dentine and also lead to pulp inflammation. Even if patients often have no clinical symptoms (pain, radiological signs of inflammation), histological examinations have shown that a chronic inflammatory reaction of the pulp is present after selective caries excavation. Consequently, the absence of clinical symptoms does not mean that the pulp tissue is healthy, nor can the actual condition of the pulp be determined using a sensibility test after selective caries excavation (free of inflammation or not). Thus, studies based on these clinical criteria are irrelevant for drawing conclusions about the success of selective caries excavation. A higher probability of success using selective caries excavation compared to vital pulp therapy (direct capping, pulpotomy) cannot be determined per se. Therefore, complete caries excavation should be favoured in order to prevent bacterial contamination and thus preclude development of a chronic inflammatory reaction of the pulp. After non-selective caries excavation, vital pulp therapy should be performed by using a calcium silicate cement for pulp capping before an adhesive restoration is subsequently placed. When performed correctly, this treatment has been shown to achieve higher success rates than selective caries excavation.

## Introduction

In addition to complete (non-selective) caries excavation, there is also the doctrine that a minimal, caries-altered dentine layer can be left in deep carious lesions. However, softened dentine and carious enamel must be completely removed. This treatment option is called ”selective caries excavation”. The aim of this procedure is to avoid exposing the pulp tissue in hope of preserving its vitality [1–3]. The idea behind selective caries excavation is to apply a tight adhesive seal over the remaining carious dentine and prohibit the caries bacteria from accessing fermentable carbohydrates. A bacteria-proof (sealed) restoration is intended to prevent adequate nutrition of the microorganisms and thus eliminate further infection. The bacteria should “starve” under the filling. The adhesive sealing of the carious dentine thus arrests the progression of caries. This method does not lead to more clinical failures than complete caries excavation. On the contrary, selective caries removal even appears to be superior to complete caries excavation [1–3].

Two systematic Cochrane reviews also came to the conclusion that selective caries excavation has clinical advantages in terms of maintaining pulp vitality and is superior to complete caries excavation. Selective caries excavation can significantly preserve pulp vitality more often. Therefore, complete removal of all caries-altered dentine should be avoided in deep carious lesions. This recommendation is evidence-based [4, 5].

In a position statement, the European Society of Endodontology (ESE) came to the conclusion that selective caries excavation is indicated in teeth having reversible pulpitis if a radiograph shows that the caries has not penetrated deeper than the last one quarter of dentine thickness toward the pulp and a residual dentinal layer separates the carious lesion from the pulp chamber [6]. The German Society of Operative Dentistry (DGZ) also favours selective caries excavation in teeth demonstrating reversible pulpitis. Carefully performed selective caries excavation is now considered the preferred excavation concept for deep carious lesions [7]. Identical recommendations can also be found in the guidelines of the Dutch Society of Endodontology (NVvE) [8].

This treatment philosophy is in contrast to the current position statement of the American Association of Endodontists (AAE) and the scientific statement of the German Society of Endodontology and Dental Traumatology (DGET). Both of these organizations generally recommend complete caries excavation to preserve the vitality of the pulp [9, 10].

The aim of this narrative review is therefore to critically examine selective caries excavation. This topic will be discussed not only from a clinical but also from a histological perspective, in order to provide recommendations for daily dental practice.

For this narrative review, many scientific databases were accessed: PubMed/Medline, Embase, Web of Science, Scopus, Cochrane Central Register of Controlled Trials (CENTRAL), and Google Scholar. Non-English and historical literature were searched manually. The period covered ranged from the first publications on caries excavation (19th century) in dental textbooks up to the year 2025.

### Historical background

From reading current publications on the subject of selective caries excavation, one can sometimes get the impression that this is a relatively new form of caries therapy. However, a random glance at historical dental textbooks shows that this is not the case. As early as the turn of the 19th and 20th centuries, in their textbooks, Andrieu (1889) [11], Miller (1896) [12], Preiswerk (1903) [13] and Sachs (1909) [14] described exactly what is known today as selective caries excavation.

Other authors, however, called for the complete removal of all carious dentine, even if this meant exposing the pulp: Koecker (1826) [15], Witzel (1886) [16], von Metnitz (1891) [17], Black (1908) [18], and Peckert (1912) [19]. The two doctrines are therefore diametrically opposed. It is therefore not surprising that Hollaender was already discussing the pros and cons of selective caries excavation as early as 1878 [20].

In the 19th and early 20th centuries one easily understandable reason for the preference for leaving carious dentine was certainly the difficulty in carrying out adequate endodontic treatment in the event of pulp exposure at that time. It was not until 1905 that procaine was developed as a local anaesthetic for dental treatment [21]. Root canal treatments were therefore very painful. Reliable concepts for cleaning, shaping, and obturating root canals were lacking. Furthermore, at the time, there were no suitable pulp capping materials to maintain pulp vitality after exposure. Hence, over 100 years ago, Rebel postulated the false doctrine that “an exposed pulp is a lost organ” [22]; a statement that unfortunately still has an effect today. It was not until a few years later that Hermann was able to prove that the pulp exposed after complete caries excavation and direct pulp capping with calcium hydroxide has a high survival rate [23, 24].

Nevertheless, in the 1940 s, Bonsack again propagated the leaving of thin carious layers under fillings and referred to this as “Coiffage Naturel” (natural pulp capping) [25, 26]. Nearly 20 years later, at the 1962 congress of the World Dental Federation (FDI) in Cologne, Germany, almost all speakers were in favour of selective caries excavation in combination with a bacteria-proof restoration for the treatment of deep carious lesions [27]. It is therefore not surprising that in 1966, Münch and Kluczka recommended selective caries excavation in their textbook, although without explicitly referring to Bonsack´s “natural pulp capping [28].”

The term “Coiffage Naturel de Bonsack” is still used today in French as a synonym for “indirect pulp capping” [29]. The term “indirect pulp capping” is also used in English to refer to the deliberate, permanent retention of slightly caries-altered dentine near the pulp under definitive restorations [30]. This terminology is in contrast to the definition in German, where indirect pulp capping is defined as covering of a thin, near-pulp, caries-free layer of dentine with a suitable medication [31]. These different definitions can lead to confusion in the interpretation of the existing international literature [32].

In the 2000 s, selective caries excavation came back into focus due to the adhesive filling technique [1, 2]. The discussion about how much caries must be removed is therefore not new and can be found again and again in the dental literature. Thus, the two opposing doctrines on caries excavation have been controversially discussed for at least 150 years.

### Clinical success rates of caries excavation

A systematic literature review showed that the probability of success of vital pulp therapy can vary considerably. This outcome is due, among other things, to the heterogeneity of the examination parameters. In most cases, success is determined on the basis of clinical and/or radiological examination criteria [33].

Using these clinical criteria, the clinical success rates for selective caries excavation reported in the literature fall from 87% after one year [34] and 83% after two years [35] to 82% after five years and to 63% after 10 years [36]. (These are exemplary results. A complete literature review can be found in [5]).

The alternative to selective caries excavation would be complete caries excavation up to pulp exposure with subsequent direct pulp capping. However, the literature on vital pulp therapy indicates that higher clinical success rates can be achieved using this technique [32]. Ricucci et al. reported that the success rate of direct pulp capping using calcium hydroxide in 225 teeth of 148 patients after 1, 5, 10, 20, and 35 years was 100%, 95%, 95%, 86%, and 89% at follow-up, respectively [37]. In another study, the success rate of direct pulp capping using calcium hydroxide was 76% after 13 years [38]. A meta-analysis concluded that the long-term clinical success rates after complete caries excavation and use of calcium hydroxide were 81.7% (± 8.9%) for indirect pulp capping, 70.1% (± 10.1%) for direct pulp capping and 79.3% (± 12.5%) for partial pulpotomy [39]. The risk of failure is significantly lower when a calcium silicate cement is used instead of calcium hydroxide for direct pulp capping [40]. Various studies have shown that long-term clinical success rates of approximately 80% are quite realistic when using calcium silicate cements for direct pulp capping after complete caries excavation, even under practice conditions (for a detailed overview see [41]).

To date, there are only a few studies that have directly compared the success rates of selective and complete caries excavation techniques. In a direct clinical comparison, teeth 10 years after selective caries excavation and adhesive restoration showed a success rate of 86%; with complete caries excavation (without pulp exposure), on the other hand, the success rate was 98% [42]. In another randomised clinical trial, the success rate for teeth clinically diagnosed as having reversible pulpitis one year after complete caries excavation and subsequent indirect or direct pulp capping with a calcium silicate cement was 98.4%. In contrast, the success rate for selective caries excavation was only 82.5% after the same timeframe. The difference was significant [43]. If complete caries excavation was followed by pulp exposure and subsequent pulpotomy, the clinical success rate after one year was 97.9%, compared to only 86% for selective caries excavation. The differences were also significant [44]. In another study, the success rate for both treatment alternatives was 95% after one year [45]. Randomized clinical trials are considered the gold standard in research methodology for evaluating the effectiveness of interventions. To date, no randomised clinical trial has been able to demonstrate a significant superiority of selective caries excavation over complete excavation.

For studies in which direct pulp capping showed significantly lower clinical success rates than selective caries excavation, errors were sometimes made during the procedure. For example, there was a lack of information on the exact procedure for direct pulp capping, no optical magnification aids (e.g. magnifying glasses) were used, no cavity disinfection was performed, a calcium hydroxide salicylate ester cement (Dycal, Kerr Life), which is unsuitable for direct pulp capping, was used (instead of a calcium silicate cement), and the cavities were initially restored provisionally instead of placing an immediate definitive restoration [46, 47]. This procedure clearly contradicts the current state of knowledge on vital pulp therapy [32]. Therefore, such incorrectly conducted studies cannot serve as evidence that selective caries excavation is superior.

### Diagnostics after caries excavation

In all studies that evaluate selective caries excavation positively, the clinical “success” was only verified by means of a sensibility test, the absence of symptoms, and/or radiological examination criteria. However, it is known that in 15.6% of cases, the clinical and histological diagnosis do not match [48]. In 14 to 60% of cases, irreversible pulpitis can be completely asymptomatic [49, 50]. The histological findings therefore often deviate from the clinical picture. Despite clinically healthy-appearing pulp (painlessness, positive sensibility test, and radiological inconspicuousness), teeth with deep carious lesions may show pronounced histological signs of inflammation in the pulp tissue. Diapedesis of leukocytes and tissue fluid, small cell infiltrations and even localised abscesses may be found. Inflammatory processes often remain confined to the crown pulp [51]. Hence, in most cases, the clinical assessment of the pulp condition is falsely positive (“hypodiagnosis”) [49, 50, 52, 53].

Histologically, there is no correlation between clinical symptoms or a positive sensibility test and the inflammatory process in the pulp [54, 55]. The accuracy and reproducibility of the usual diagnostic tests for assessing the vitality of the pulp (sensibility test) are therefore limited or even inadequate. To date, there is a lack of reliable methods to clinically determine the actual status of the pulp [56].

It is therefore too short-sighted to merely consider the absence of clinical symptoms after selective caries excavation as a successful treatment. The patients’ clinical responses to the sensibility test may not correlate with the histological findings. After selective caries excavation, histologically visible chronic inflammation, microabscesses, and necrosis may occur without patients experiencing any symptoms; in other words, the pulp may be irreversibly inflamed after selective caries excavation without the patient noticing. In 81 of 224 teeth (36%), no pain occurred despite partial necrosis of the pulp and a pronounced inflammatory reaction [57]. Despite clinical freedom from symptoms and positive sensibility testing, after selective caries excavation inflammatory cells were found histologically in the pulp and granulocytes as part of the cellular immune defence in the afferent blood vessels in all of the 12 teeth examined 1 to 9 months [58].

It is therefore wrong to assume that if there are no symptoms or the sensibility test is positive, the selective caries excavation was successful [55]. The absence of pain or a positive sensibility test does not mean the absence of inflammation or necrosis of the pulp [59]. All these findings are not new, but have been scientifically proven for almost 100 years [60].

### Histological results after caries excavation

**Fig. 1:**
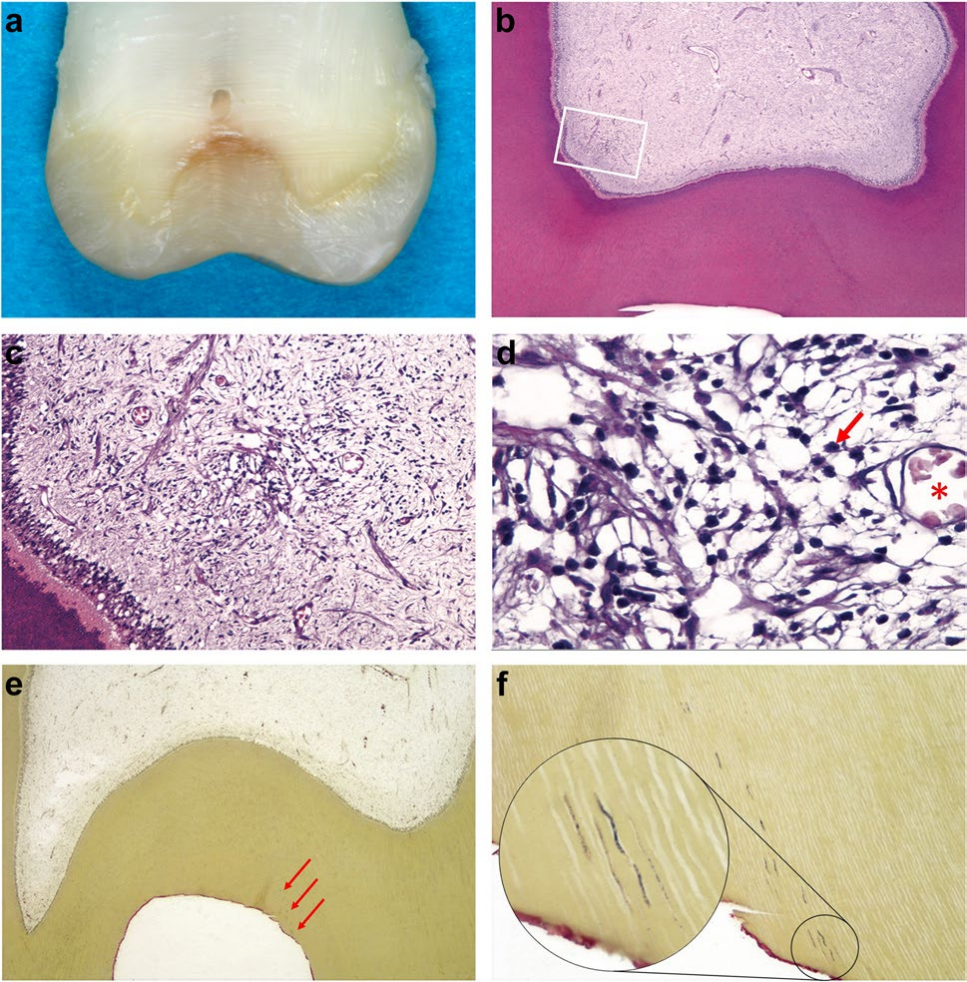
Tooth 18 of a 38-year-old male patient having deep occlusal caries and scheduled for extraction. The patients gave consent to have caries removed selectively and the cavity restored with adhesive procedures prior to extraction. A selective caries excavation was performed, leaving firm dentine in the cavity floor. The cavity was restored using a dentine adhesive and resin composite. No symptoms occurred during the post-operative period. The tooth was extracted after two months. (Unpublished case from [58]). **a** The tooth with occlusal resin composite restoration was ground on a bucco-lingual plane to enable fixation of the pulp and alignment of the specimen in the paraffin block. **b** View of the pulp chamber at low magnification. Histological slides were stained with haematoxylin and eosin (H&E) for evaluation of pulp changes (original magnification ×16). **c** Middle magnification of the area delimited by the rectangle in Fig. 1b. A concentration of cells can be seen in the subodontoblastic area (original magnification ×100). **d** High magnification of Fig. 1c shows that these cells are lymphocytes. For example, a lymphocyte is marked with a red arrow. The red asterisk indicates a pulpal capillary filled with erythrocytes. (original magnification ×400). **e** Bacterial staining after selective caries excavation. Even at this low magnification, bacteria can be suspected in the dentinal tubules. See red arrows. (Taylor modified Brown & Brenn, original magnification ×16). **f** Detail from the cavity floor confirms the suspicion of bacterial presence. The dentinal tubules are colonised by bacteria to a considerable depth. The red-coloured line on the cavity floor is the hybrid layer (original magnification ×100, inset ×1000). Images and interpretations courtesy of Dr. D. Ricucci (Cetraro, Italy) and Prof. Dr. M. Lipski (Szczecin, Poland)

Because a clinical assessment is not sufficient to prove the success of selective caries excavation, it seems sensible to histologically examine the effect of leaving caries close to the pulp. Histological examination showed that selective caries excavation with subsequent definitive restoration of the teeth leads to inflammatory alterations in the pulp (irreversible pulpitis) in 68 to 100% of cases. In contrast, after complete caries excavation and subsequent indirect or direct pulp capping using calcium hydroxide, histological failure occurred in only 7% (indirect pulp capping) to 33% (direct pulp capping). In all cases, the patients were clinically free of symptoms for between 3 months and 5 years during the entire follow-up period. The sensibility test was positive in all cases [61, 62]. After selective caries excavation, chronic inflammatory cell infiltrates were identified histologically in the pulps of all 12 teeth examined. After 1 to 9 months, both scattered inflammatory cells and extensive localised accumulations of inflammatory cells were detected. Pulpal capillaries were clearly filled with erythrocytes and polymorphonuclear leucocytes. A large number of stainable bacteria was observed in the dentine below the cavity floor in all samples. The bacteria remaining in the dentine provoked a subclinical pulp inflammation over the entire examination period. All patients were therefore clinically completely free of symptoms and the sensibility test was positive. An ex-vivo example of remaining bacterial presence within dentine tubules and the pulp’s response are presented in the series of photographs displayed in Fig. 1. This tooth had to be extracted after receiving selective caries removal treatment after a 2-months period of placement. (Fig. 1a-f) Selective caries excavation does not lead to control or even disappearance of the bacterial infection [58]. Degeneration or inflammation of the pulp tissue after selective caries excavation is the rule rather than the exception [55].

### Caries-infected and caries-altered dentine

Some authors differentiate between caries-infected and caries-altered dentine in a caries lesion. Accordingly, the outer carious layer is infected with bacteria (*caries-infected dentine*), which leads to a non-remineralisable necrotic collagen matrix. This dentine must be removed. In the inner, underlying layer, bacteria are not observed at all or much less frequently (*caries affected dentine*). This dentine can be left in place, even if it is softened (leathery) [63, 64].

However, if samples are taken from this caries-altered dentine layer under fillings, histological and bacteriological studies have shown that bacteria can always be detected and that these microorganisms are capable of multiplying [54, 55, 58, 65]. Viable microorganisms could still be recovered from the caries-altered dentine layer long after a restoration had been placed [65]. Bacteria survive under fillings and lead to irreversible pulpitis [55]. If only the superficial caries-infected dentine layers are removed, it is highly probable that a considerable number of bacteria capable of reproduction will be left behind in the caries-altered dentine layer [54]. The idea that there is a caries-altered dentine layer that is more or less free of microorganisms is therefore a myth and incorrect.

Furthermore, bacteria are also found in the dentinal tubules below the caries-altered dentine layer, which diffuse towards the pulp (Fig. 1e and f) [54, 55, 58]. Bacterial colonization of the dentinal tubules is of crucial importance for the progression of caries and the development of pulpal and periapical inflammation. Due to the unique location of the bacteria in the dentinal tubules during the carious process, the body’s own defence mechanisms cannot become active there. Phagocytosis (killing of microorganisms) by defence cells does not occur until the pulp tissue is in direct contact with the caries bacteria [66–68].

### Microorganisms and endotoxins

In principle, it is questionable whether it is actually always clinically possible to place bacteria-proof fillings. Especially in the proximal region, it is virtually impossible to clinically verify the seal. The marginal seal of fillings after selective caries excavation is therefore not guaranteed in the long term [69].

Regardless of whether a filling is bacteria-tight or not, and in addition to cariogenic bacteria that feed on carbohydrates, certain anaerobic asaccharolytic bacteria that use nitrogenous substrates for energy production are also found in infected dentine. Proteins and glycoproteins from demineralised collagen and tissue fluid from the dentinal tubules serve as nutrition sources for these microorganisms. Anaerobic asaccharolytic bacteria can also multiply under “sealed” fillings. The degradation products of these microorganisms then lead to inflammation of the pulp tissue [70–73].

The application of calcium hydroxide does not lead to disinfection of the cavity either. Following selective caries excavation and application of calcium hydroxide for eight weeks, acidogenic and aciduric streptococci and lactobacilli were limited in their ability to survive, but actinomycetes and streptomycetes, which prefer the alkaline environment, were favoured [74]. These bacteria can survive in carious dentine under fillings. Bacteria remaining in the dentine therefore always harbour the risk of caries recurrence and form a reservoir of toxins (endotoxins) even after the microorganisms have died, which can maintain inflammatory processes in the pulp [75]. If these bacterial metabolites (endotoxins such as lipopolysaccharides and lipoteichoic acids) are released in deep cavities, they diffuse through the remaining dentine and penetrate into the pulp. As a result, inflammatory mediators are released from the odontoblasts and macrophages, which lead to chronic pulpal inflammation [76]. This condition means that even if bacteria do not survive under filling and die, inflammatory reactions in the pulp still occur. This reaction does not require the bacteria themselves to penetrate the tissue: Just metabolic products are sufficient. Ultimately, it is therefore irrelevant whether the microorganisms “starve” under a filling or not.

Both bacteria and bacterial metabolic products from caries (endotoxins) can diffuse through dentinal tubules towards the pulp and then cause an inflammatory change in pulp tissue [77, 78]. Microorganisms can penetrate 500 to 3,000 μm deep into the dentine of pulp-vital teeth towards the pulp within 7 weeks to 8 months [79, 80]. Endotoxins easily diffuse through 0.5 mm thick dentine [81]. Therefore, inactive caries also leads to histological changes in the pulp tissue [82], but this often takes years [55].

A pronounced layer of irritant, secondary dentine on the cavity floor does not protect the pulp from carious bacteria nor from their metabolic products and thus does not protect the pulp from necrosis caused by caries [83, 84]. Even without exposure of the pulp tissue, inflammation occurs due to bacterial decomposition products (endotoxins). Pulp reactions already occur as a result of superficial dentinal caries. There is a direct correlation between the extent of the caries and the pulp’s reaction to it. As soon as the dentine caries spreads towards the pulp, the pulp reactions increase. It is not the entire pulp that is inflamed, but only the areas where bacteria or endotoxins penetrate [58, 62, 83]. In this area, the pulp loses its ability to regenerate (transition from reversible to irreversible pulpitis) [66–68]. In contrast, a reduction in inflammation can be demonstrated histologically if the entire carious dentine is removed [83].

Microorganisms and their metabolic products play a decisive role in the development of pathogenic pulpal changes and periapical diseases. The presence or absence of microorganisms is the determining factor in the furtherance of disease or a transition to healing of pulp tissue [85, 86].

### Monomers from filling materials

The components of dentine adhesives and resin composite materials pose another problem if they are applied directly to the dentine in a deep cavity after selective caries excavation without subbase. For example, in a scientific communication, the German Society of Operative Dentistry (DGZ) recommends that in the case of indirect pulp capping, the dentine above the pulp should not be treated with medication, as there is no evidence of any benefit [7]. However, if this approach is taken, it is important to be aware that unpolymerized components of dentine adhesives and resin composites cause immunosuppression of the pulp’s defence cells and limit the pulp’s natural defence capacity [84, 87]. If carious, infected dentine remains near the pulp, in addition to the bacterial metabolic products (endotoxins), composite components (e.g. monomers) are also released, which also diffuse through the thin residual dentine and penetrate into the pulp. This movement causes additional damage to the pulp tissue [76]. Dentine adhesive components can easily penetrate thin layers of dentine. Although caries leads to dentine sclerosis, TEGMA and HEMA, for example, also diffuse through sclerosed dentine [88]. Pulp necrosis may therefore occur as a result of the cumulative effects of residual microorganisms from selective caries excavation in combination with leachates from pulp-toxic restorative materials [89].

### Filling fractures after selective caries excavation

A basic prerequisite for the success of selective caries excavation is that the carious lesion is effectively isolated from the oral environment by the hermetically sealed, adhesive restoration. However, the adhesion of dentine adhesives and composites to caries-altered dentine is significantly lower compared to adhesion to caries-free dentine [90–93]. Therefore, teeth after selective caries excavation and dentine-adhesive restoration show a highly significant lower fracture resistance of resin composite restorations compared to fully excavated, restored teeth. Fractures within resin composite restorations and microleakage occurred significantly more frequently [94]. If microorganisms penetrate under the filling via this fracture gap or if surviving bacteria gain access to nutritive substrates via penetrating saliva, they continue their dentine-dissolving process underneath the hybrid layer. In this case, the hybrid layer is destroyed and the dentine collapses and separates, causing a gap to form between the filling and the dentine. Further bacteria can penetrate via this microleakage pathway and lead to pulp degeneration.

## Discussion

It should be noted that this narrative review is a qualitative literature review that summarises and classifies existing research results without following a strictly systematic approach. The author uses a narrative structure to create a broad, context-related presentation of the topic and to highlight new interpretations and gaps in the research.

The main criticisms of conventional, complete caries excavation can be summarised as follows: There are sufficient evidence-based clinical studies that demonstrate success rates for selective caries excavation that are equal, if not superior, to those using complete, non-selective caries excavation [5]. Complete caries excavation is therefore not necessary. All these studies on selective caries excavation (for an overview, see [5]) have certainly been performed very carefully and with great scientific commitment. The problem with these clinical studies, however, is that it is not known what the condition of the pulp actually was at the time of tooth assessment. Of course, no statement can be made about this aspect in a clinical study. However, it has been sufficiently proven that there is no good correlation between clinical freedom from symptoms and a positive sensibility test on the one hand and the actual condition of the pulp on the other [56]. This concept means that the pulp may be bacterially infected without the patient realising it. Well-founded prognostic indicators that allow a reliable assessment of the outcome of treatment even after selective caries excavation are currently not available [56]. These results have been incorporated into the current S3 guidelines of the European Society of Endodontics (ESE) [95].

In order to assess the actual condition of the pulp, histological studies must be carried out under scientific aspects. This inclusion is the only way to obtain certainty about the treatment outcome. All histological studies cited in this narrative review come to the clear conclusion that leaving caries in the sense of selective caries excavation sooner or later leads to irreversible pulpitis [54, 55, 58, 59, 61, 62, 65, 83, 89]. Conversely, there is not a single study to date that has been able to prove that selective caries excavation does not lead to pulpal changes. However, conducting such histological studies on humans is time-consuming and must always be considered from an ethical point of view, as the teeth inevitably have to be extracted for this purpose. The number of such studies is therefore unfortunately limited and will remain so.

Proponents of selective caries excavation consider the preservation of pulp vitality to be successful if the patient has no complaints, the tooth functions perfectly and no radiological abnormalities are visible. The majority of patients who have undergone selective caries excavation are clinically symptom-free. However, from a medical point of view, being free of symptoms does not mean that there is no need for treatment. Typical examples in dentistry are gingivitis and periodontitis. Even periapical periodontitis does not necessarily have to be associated with symptoms. However, this does not mean that these diseases are left untreated. The same applies in human medicine: Widespread diseases such as hypertension or diabetes also cause no clinical symptoms, at least in the early stages. Nevertheless, they require treatment. Adenomas discovered during a preventive colonoscopy must also be removed, even if they do not cause any health problems. Histological examinations have sufficiently demonstrated that adenomas can be precursors to carcinomas. All of these conditions can cause significant health problems later on if they are not treated early. The absence of symptoms does not mean that the tissue is healthy and that the therapy was therefore successful. This relationship exists because symptom-free patients can have an “ongoing” chronic inflammation that is harmful to them. In the medical sense, however, treatment can only be considered successful if there are no abnormalities from a histopathological point of view.

The negative consequences of leaving microorganisms behind during selective caries excavation are also considered negligible. However, leaving carious dentine over the pulp is analogous to leaving bacteria or colonising bacteria in the vicinity of a surgical wound, which can then maintain inflammation and lead to necrosis [62]. Therefore, in accordance with generally accepted surgical principles, carious dentine must be removed completely. This is the only way to restore the disturbed biological balance in the pulp [96].

No treatment concept is known from human medicine in which an infected, pathologically altered body tissue is left in place, although it can technically be removed without any problems. Technically, complete caries excavation and pulp capping are not difficult if you know how to perform the treatment correctly. Therefore, it is questionable if the fear of pulp exposure really results from the dental clinicians´ unfamiliarity with performing the next steps with competence, should an overt pulp exposure occur. Root canal treatments, telescopic denture or implants are certainly more technically complex and error-prone, but are nevertheless carried out by “general dentists” with great success.

There is clear histological evidence of a direct correlation between bacteria remaining in the cavity under a restoration and the presence of pulp inflammation. From a histological point of view, leaving caries under a filling in the sense of selective caries excavation cannot be recommended, as this has been shown to lead to chronic, subclinical inflammation of pulp tissue. In order to preserve the pulp vitality for the long term, the caries-altered dentine should be completely removed [54, 55, 58, 59, 61, 62, 65, 83, 89].

For all these reasons, both the American Association of Endodontists (AAE) and the German Society of Endodontology and Dental Traumatology (DGET) recommend complete caries excavation in their current scientific statements on vital pulp therapy [9, 10]. This is the only way to clinically assess the actual condition of the pulp tissue. If carious dentine remains in a deep carious lesion, this obstructs the view of possible pulp changes such as hyperaemia or necrosis. There is also a risk of overlooking existing minimal pulp exposure if a thin layer of carious dentine remains at the deepest part of the cavity. Furthermore, there is no scientific evidence that the currently propagated selective caries excavation has a higher probability of success compared to vital pulp therapy after pulp exposure [9, 10].

Despite clear histological evidence that selective caries excavation leads to chronic, subclinical inflammation of the pulp, the ultimate question is what one wants to achieve with its treatment. Selective caries excavation probably leads “only” to a lack of clinical symptoms after treatment, but not to histologically healthy pulp tissue in the long term. If, on the other hand, complete caries excavation is performed, a high percentage the pulp tissue remains histologically healthy even after direct pulp capping or partial pulpotomy. To successfully preserve the vitality of the pulp for the long term, the caries should therefore also be completely excavated also in deep cavities. Finally, the cavity must be disinfected (sodium hypochlorite) and the pulp-dentine complex treated with a suitable capping material (calcium silicate cement) [32]. The aim of dental medicine should be to heal a tooth, a tissue or even the pulp as far as possible and not just to achieve clinical freedom from symptoms by leaving chronic inflammatory tissue in place.

Because pulpal changes generally occur slowly when caries is left under fillings [55], selective caries excavation may be indicated when the clinical treatment situation is difficult. Such situations include teeth having only a limited retention time in the mouth, e.g. in very young patients with primary dentition or very old patients with underlying diseases.

## Conclusion

In deep carious lesions, complete caries excavation and capping of the pulp with a calcium silicate cement has been shown to result in clinically significantly higher survival rates compared to selective caries excavation. Exposure of the pulp tissue during caries excavation can therefore not be regarded as a negative prognostic factor for pulp vitality. There is also evidence of bacterial survival after selective caries excavation under restorations, which histologically causes inflammation of the pulp tissue significantly more frequently. Hence, in deep carious lesions of permanent teeth, complete, non-selective caries excavation down to hard dentine is recommended to ensure predictable long-term pulp survival.

## Data Availability

No datasets were generated or analysed during the current study.
